# Rapid assessment of fern spore quality using microscale electrical conductivity testing

**DOI:** 10.1002/aps3.70078

**Published:** 2026-09-09

**Authors:** Yao‐Moan Huang, Chia‐Wen Ko, Chin‐Tzer Duh, Chu‐Mei Huang, Wen‐Liang Chiou, Tzu‐Tong Kao

**Affiliations:** ^1^ Forest Ecology Division Taiwan Forestry Research Institute No. 53, Nanhai Road Taipei City 100060 Zhongzheng District Taiwan; ^2^ Silviculture Division Taiwan Forestry Research Institute No. 53, Nanhai Road Taipei 100060 Zhongzheng District Taiwan; ^3^ Herbarium Taiwan Forestry Research Institute No. 53, Nanhai Road Taipei 100060 Zhongzheng District Taiwan; ^4^ Department of Biological Sciences National Sun Yat‐sen University 70 Lienhai Road Kaohsiung 804201 Gushan District Taiwan

**Keywords:** chlorophyllous spores, ex situ conservation, germination, longevity, *Plenasium banksiifolium*, Polypodiophyta, spore viability, spore vigor

## Abstract

**Premise:**

Ex situ spore banks are essential for fern conservation and research, yet traditional germination tests for assessing spore quality are time consuming and labor intensive. We explored whether electrical conductivity (EC), a rapid proxy for seed vigor, could offer an efficient alternative for evaluating fern spore quality.

**Methods:**

Using chlorophyllous spores of *Plenasium banksiifolium*, we developed a microscale EC protocol (0.01‐g spores, 1‐h immersion) to track EC and germination over eight weeks of storage. Spore longevity (*p*_50_) was estimated via probit analysis, and correlations among EC, viability, and longevity were assessed.

**Results:**

Fresh spores from different individual plants showed varied initial EC (EC_0_) values, with lower EC_0_ values associated with longer longevity (*p*_50_). During storage, EC values increased over time, while germination percentage declined. The relative EC ratio (EC_i_/EC_0_) showed significant negative correlations with germination percentage, with a ratio of ~2 indicating 50% viability loss and ~3 signaling complete viability loss.

**Discussion:**

The EC test presents a promising approach for rapidly assessing spore viability and longevity. Lower initial EC (EC_0_) values predicted greater initial spore viability and longer longevity, likely reflecting differences in spore maturity. The relative EC_i_/EC_0_ ratio effectively tracked viability loss, supporting its use in improving spore bank management.

Ferns (Polypodiopsida) are the second most species‐rich group of extant vascular plants, with only angiosperms having a greater number of species (Sessa et al., 2014). Unlike seed plants, their gametophytes are free‐living and develop independently from the sporophyte. This unique life cycle enables ferns to disperse via spores rather than seeds (Brock, 2025). These spores are single‐celled propagules produced through meiosis on the fertile sporophytic fronds (sporophylls). Due to the small size of spores and their high potential for long‐term storage, spore banking has become an increasingly important tool in ex situ conservation, offering a compact and cost‐effective approach of preserving fern biodiversity, while supporting research, horticulture, and ecological restoration (Dyer, 1994; Pence, 2004; Ballesteros, 2008). In recent decades, efforts have focused primarily on optimizing storage conditions to maintain spore viability (e.g., Ballesteros and Walters, 2011; Ballesteros et al., 2012, 2019). However, as spore collections grow, the ability to efficiently assess spore quality at scale has emerged as a critical challenge for spore bank management.

Current spore viability assessment relies heavily on germination tests, where spores are incubated under controlled conditions and monitored for rhizoid emergence. Although these tests are direct and reliable, they are labor intensive, requiring weeks of observation by trained personnel (Lloyd and Klekowski, 1970; Raghavan, 1989; Hoekstra et al., 2001; Ballesteros and Walters, 2011), making them impractical for high‐throughput or rapid assessments in spore banks. Moreover, certain taxa, such as those in Ophioglossaceae and Psilotaceae, require mycorrhizal symbiosis for germination (Lehnert and Kessler, 2016), limiting the efficacy of standard cultivation‐based assays.

In addition to viability, spore longevity—the ability to germinate following storage—is also valuable for effective spore bank management. In seed biology, longevity is a component of vigor, alongside germination uniformity and performance under unfavorable conditions (Hampton and Tekrony, 1995). The International Seed Testing Association (ISTA) recognizes several vigor assessment methods, including electrical conductivity (EC) testing—one of the earliest ISTA‐endorsed techniques for seed vigor testing—which assesses membrane integrity through measurements of electrolyte leakage during immersion (ISTA, 2025). High‐vigor seeds exhibit lower EC values due to effective membrane repair, enabling early detection of deterioration before germination declines (Matthews and Powell, 2006; Demir et al., 2012; Batista et al., 2021). However, comparable methods for fern spores remain underdeveloped. Beyond germination tests, only the tetrazolium chloride (TTC) assay has been explored, in which batches of 10^4^–10^5^ spores of *Dryopteris guanchica* Gibby & Jermy were stained and absorbance measured to infer mitochondrial activity as an ecotoxicity index (Catala et al., 2009; Gabriel y Galán and Prada, 2010). While effective for ecotoxicity assessments, this approach does not assess the actual germination capacity of spores, highlighting the need for conservation‐oriented vigor assessment techniques (Chang et al., 2015; Pedrero‐López et al., 2021).

Fern spores can be broadly classified into two physiological types: chlorophyllous (green) and non‐chlorophyllous (non‐green). Chlorophyllous spores occur in approximately 1400 species (ca. 14% of total fern diversity), have evolved independently multiple times, and are overrepresented in fern lineages adapted to epiphytic and waterlogged environments with limited opportunities for mycorrhizal associations, such as Equisetaceae, Osmundaceae, Hymenophyllaceae, Onocleaceae, and grammatids (Polypodiaceae) (Sundue et al., 2011; Mellado‐Mansilla et al., 2021, 2022, 2025). They are photosynthetically active but short‐lived, often losing viability within weeks (Lloyd and Klekowski, 1970; Ballesteros et al., 2017). Their rapid decline in viability makes them particularly suitable for testing new vigor assessment methods. Previous studies on *Pentarhizidium orientale* (Hook.) Hayata demonstrated that the viability and longevity of chlorophyllous spores vary significantly with maturity—an attribute that cannot be reliably inferred from spore morphology or initial germination percentages (Huang et al., 2024a). *Plenasium banksiifolium* (C.Presl) C.Presl (Osmundaceae) produces abundant spores on its dimorphic fertile pinnae, making it a practical system for assay development. Our preliminary observations in *P. banksiifolium* revealed a similar maturity‐dependent pattern, underscoring the need for a reliable method to assess spore viability and longevity.

Here, we present the first application of the EC test for fern spores, using *P. banksiifolium* as a model. Our objectives were to (1) establish a microscale EC protocol suitable for fern spores, which are characterized by their small size (typically 30–100 µm in diameter) and light weight (approximately 15–30 million spores per gram); (2) compare EC reading with conventional germination test results across a storage period of 0–8 weeks; and (3) assess the correlation between EC, germination percentage, and longevity to evaluate the predictive value of EC as a proxy for spore viability and vigor.

## METHODS

### Spore collection

Fully expanded fertile fronds of five *P. banksiifolium* plants cultivated in the greenhouse of the Dr. Cecilia Koo Botanic Conservation Center, Pingtung, Taiwan, were collected in March 2024 (Figure 1). To facilitate spore release, each frond was air‐dried for three days on white paper at 50% relative humidity and 25°C. Voucher specimens have been deposited in the herbarium of the Taiwan Forestry Research Institute (*TAIF 578314*, *578315*, *578318*, *578319*, *578322*, *578323*).

**Figure 1:**
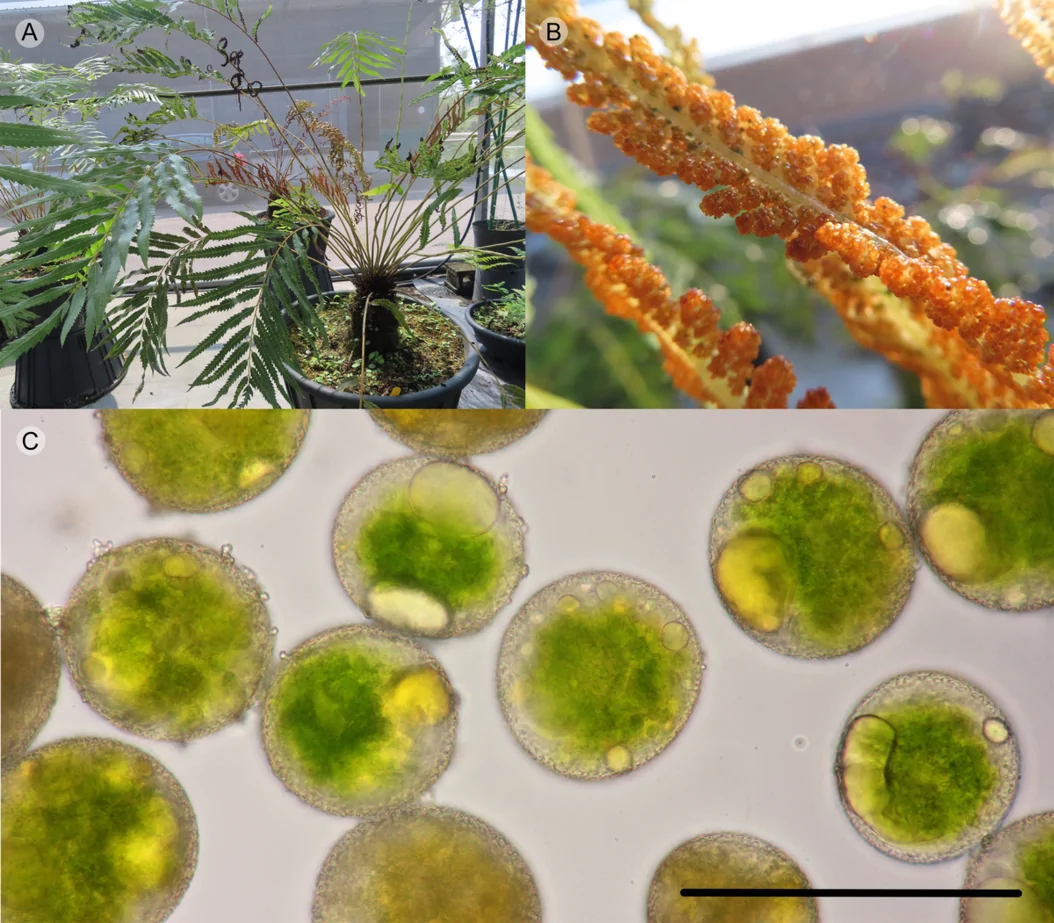
Morphology of *Plenasium banksiifolium* and its spores. (A) Greenhouse‐grown plants at the Dr. Cecilia Koo Botanic Conservation Center, Pingtung, Taiwan. (B) Fertile pinna bearing numerous sporangia. (C) Chlorophyllous spores released from fertile pinnae. Scale bar = 100 μm.

### Determination of spore mass and immersion duration for EC testing

To determine the optimal spore mass and immersion duration for EC testing, spores from plant #5 (which yielded the highest spore mass) were used. Spore masses of 0.001, 0.005, 0.01, 0.05, and 0.1 g were sampled and measured using a microbalance with 0.0001‐g precision (Phoenix GH‐202 Analytical Balance; A&D Co., Tokyo, Japan). Each sample was placed in a 1.5‐mL microcentrifuge tube and 1 mL of distilled water was added. The tubes were gently shaken for 10 s, and the samples were left immersed for 0, 0.25, 0.5, 1, 2, 4, 8, or 24 h before testing. After each soaking period, the suspension was gently mixed, and 0.3 mL of the solution was extracted and measured, avoiding the bottom of the tube to minimize potential contamination from settled debris. EC was measured using a handheld EC meter (LAQUA Twin EC Meter; HORIBA Advanced Techno, Tokyo, Japan), which requires only a minimum volume of 0.12 mL.

### Serial EC measurements

Based on the optimization results described above, a combined condition of 0.01 g of spores (approximately 150,000 spores, estimated via serial dilution) from plant #5 and a 1‐h immersion duration were selected for EC testing. This spore mass–time combination balanced sensitivity, stability, and efficiency while accounting for their interaction, as smaller spore masses reached EC saturation more rapidly (Figure 2; detailed in the Results section under “Optimization of the EC test protocol”). Spores from plants #1–4 did not differ significantly in mean spore diameter (62.0 ± 3.5, 62.3 ± 3.4, 61.7 ± 3.5, and 62.7 ± 3.3 μm in plants #1–4, respectively; mean ± standard deviation [SD], *n* = 50, one‐way ANOVA, *p* = 0.509). Spores from each plant were subdivided into nine 1.5‐mL microcentrifuge tubes (0.01 g per tube) and stored at 25°C ± 3°C in darkness. EC measurement and germination testing were performed weekly on one subsample from each plant. EC_0_ refers to the electrical conductivity value measured after the standardized 1‐h immersion at the start of the experiment (week 0), prior to storage, and represents the baseline electrolyte leakage of each spore source (plants #1–4).

**Figure 2:**
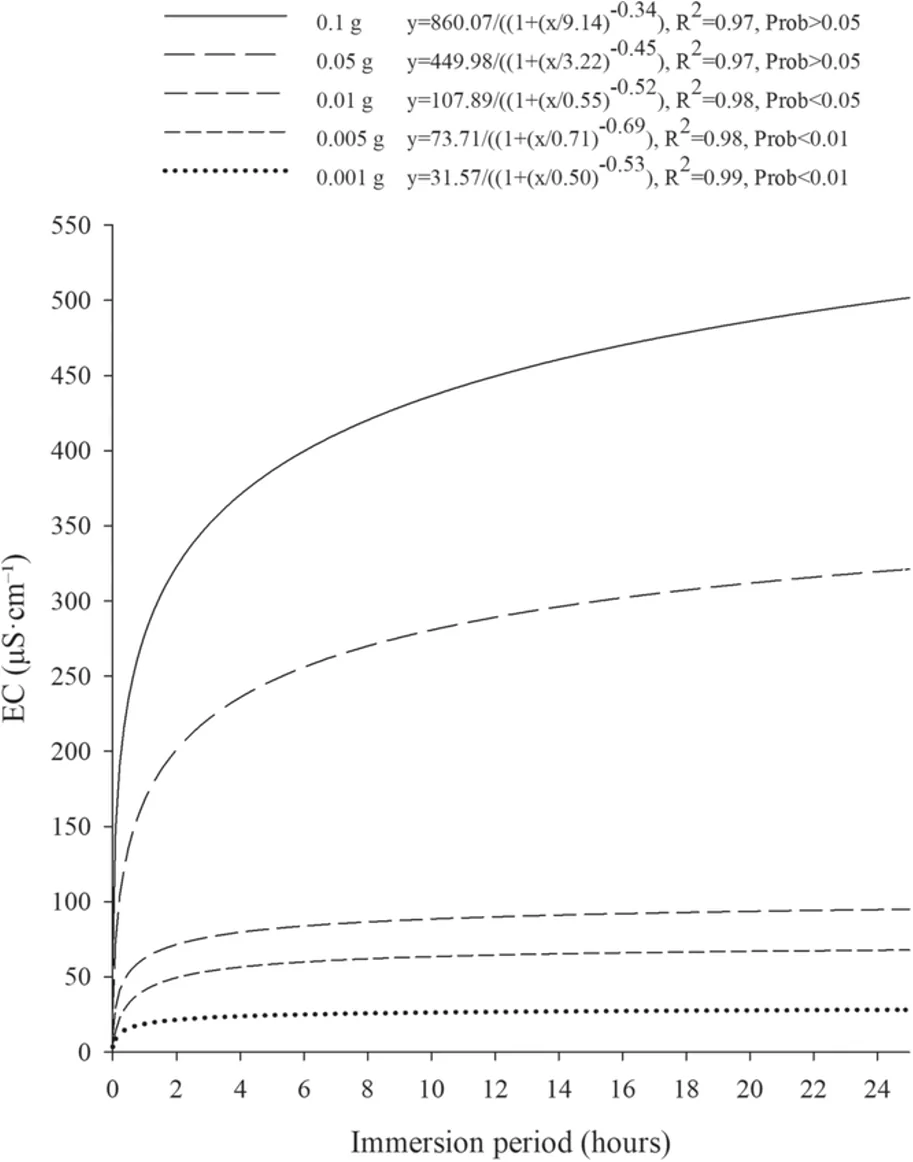
Electrical conductivity (EC) curves of *Plenasium banksiifolium* spores across different sample masses. EC values (μS·cm^−1^) were recorded during immersion of spores of five different masses (0.001, 0.005, 0.01, 0.05, and 0.1 g) over a 24‐h period. Lines indicate fitted regression curves of measured values. Larger sample masses (0.05 and 0.1 g) produced higher EC readings and slower equilibration, while smaller masses stabilized within 1–2 h.

### Germination testing

Following EC measurement, the remaining spore suspensions were pipetted onto a 47‐mm segment of filter membranes (Supor 450, 0.45‐µm pore size; Pall Co., Ann Arbor, Michigan, USA) placed in 90‐mm Petri dishes (BL 6905; Violet BioScience, Taipei, Taiwan). Six 70‐mm segments of absorbent membrane (Toyo Roshi Kaisha, Tokyo, Japan) pre‐soaked with 5.5 ± 0.5 mL of cooled, boiled water were laid under each 47‐mm membrane to maintain humidity. Dishes were sealed and incubated under 12‐h light/dark cycles (30 µmol·m^−2^·s^−1^ photosynthetic photon flux density; LED white light) at 23–25°C. Four replicates were prepared per spore source (plants #1–4). Germination percentages were recorded weekly for four weeks by counting 50 spores per replicate using a dissecting microscope (Leica Wild M8; Leica Microsystems, Wetzlar, Germany) and photographed with a Canon 550D camera (Canon, Tokyo, Japan). Germination was defined as the emergence of the first rhizoid. Data are expressed as mean ± SD (*n* = 4) unless otherwise stated.

### Calculation of germination percentage and longevity

The best‐fitting curves of germination percentage and EC over storage time were plotted using SigmaPlot 14.0 software (Systat Software, San Jose, California, USA). Spore longevity (*p*_50_) was estimated via probit analysis. Germination percentages (in probit units) were regressed against storage duration to estimate the rate of decline. The equation used for probit analysis followed Ellis and Roberts (1980):

$$v=K_{i}\;–\;p/\sigma$$

Where *v* is the probit‐transformed germination percentage after *p* weeks of storage; *K*_i_ is the probit‐transformed initial germination (*y*‐intercept); and σ is the time for germination to decrease by 1 probit, with the slope (1/σ) indicating the deterioration rate (Obunyali et al., 2008). When *v* = 0 (i.e., 50% germination), *p*_50_ is calculated following Ellis and Roberts (1980):

$$p_{50}=K_{i}\times \sigma$$

## RESULTS

### Optimization of the EC test protocol

The EC test was optimized for *P. banksiifolium* spores using varying spore masses and immersion times (Figure 2, Appendix S1). Initial EC (EC_0_) was undetectable at 0.001 g but measurable at ≥0.005 g. EC values increased rapidly within the first hour of immersion, reaching 18, 41, 66, 188, and 301 μS·cm^−1^ for 0.001, 0.005, 0.01, 0.05, and 0.1 g, respectively. For smaller sample masses (<0.01 g), EC values approached a steady level after 1 h of immersion. In contrast, samples with larger spore quantities (0.05–0.1 g) continued to show upward trends in EC values throughout the 24‐h period. This slower progression toward equilibrium may result from a reduced osmotic pressure gradient in these samples, where higher solute concentrations in the immersion medium slow the rate of electrolyte leakage from the spore cytoplasm.

A key objective of adapting the EC test is to provide a rapid, low‐input alternative to conventional germination tests for evaluating spore quality. Therefore, minimizing both spore quantity and immersion time is preferred. However, very small spore masses (≤0.005 g) produced inconsistent readings due to limitations in microbalance precision, making 0.01 g the recommended minimum. Regarding immersion time, most electrolyte leakage occurred within the first hour, with EC values of samples with smaller mass approaching equilibrium thereafter. Prolonged soaking increases the risk of immersion‐induced damage; thus, an immersion period of 1–4 h is recommended to ensure sensitivity while preserving spore integrity.

### Spores from different sources exhibit distinct EC trends

EC measurements for *P. banksiifolium* spores varied considerably among the four sources in both EC_0_ and subsequent increasing patterns, although final fold changes after eight weeks of storage were relative consistent, ranging from 2.79 to 3.90 (Figure 3, Appendix S2). At storage initiation, spores of plant #1 exhibited the lowest EC_0_ (21.0 ± 1.4 μS·cm^−1^), increasing steadily to 78.75 ± 2.75 μS·cm^−1^ after eight weeks of storage (a 3.75‐fold increase). Spores of plants #2 and #4 started with similar EC_0_ values (34.75 ± 0.96 and 34.25 ± 0.50 μS·cm^−1^, respectively) and plateaued after six weeks of storage at 97.00 ± 2.58 μS·cm^−1^ (2.79‐fold increase) and 133.50 ± 3.32 μS·cm^−1^ (3.90‐fold increase), respectively. Spores of plant #3 had the highest EC_0_ (101.75 ± 3.78 μS·cm^−1^), increasing rapidly to a plateau of 291.75 ± 3.59 μS·cm^−1^ (2.87‐fold increase) within three weeks of storage.

**Figure 3:**
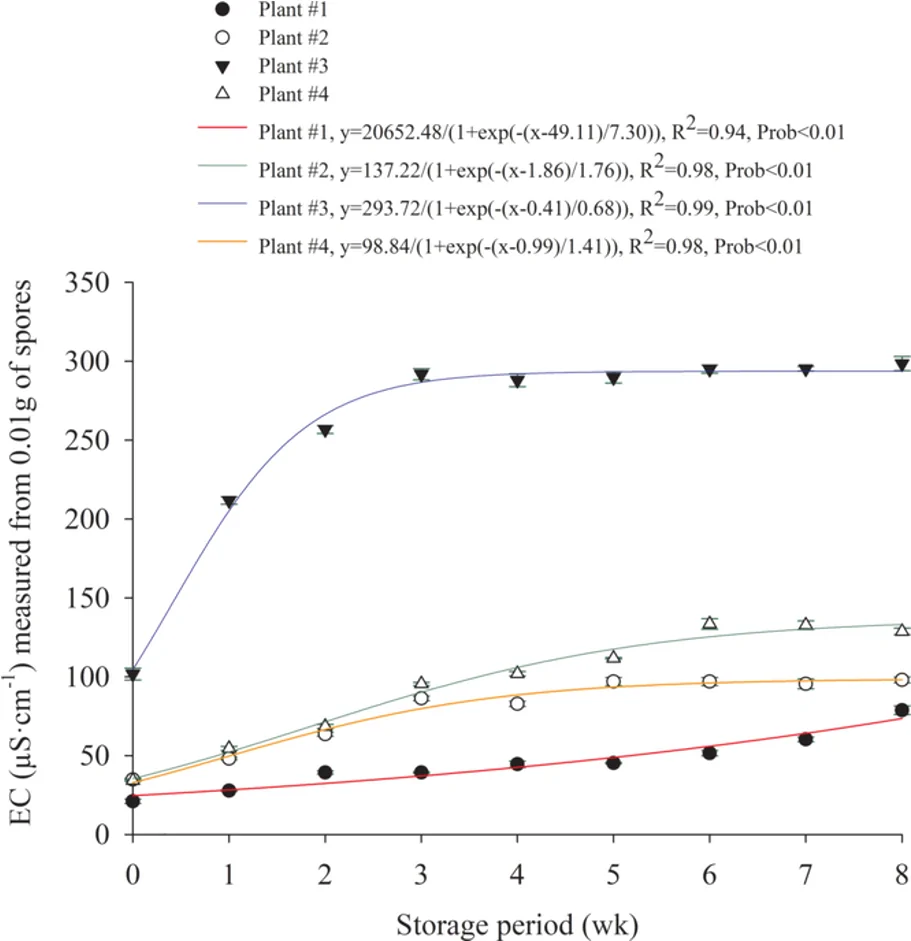
Electrical conductivity (EC) curves of *Plenasium banksiifolium* spores from four individual plants after different storage periods. The initial EC values (EC_0_) of a sample from each plant were measured at week 0, and the subsequent EC values (EC_i_) were measured from weekly subsamples over an 8‐week storage trial. Symbols indicate different plant sources, and lines show fitted regression curves. Error bars represent standard deviation (*n* = 4 spore subsamples per plant). Statistical results of one‐way ANOVA followed by Tukey's honestly significant difference (HSD) test (*p* < 0.05) are shown by letters: uppercase letters indicate significant differences among plants in EC_0_ (week 0) and lowercase letters indicate significant differences in EC after eight weeks of storage.

### Spores from different sources also exhibit different longevity

Germination percentage curves followed a reverse sigmoid or reverse J‐shaped pattern over the 8‐week storage period (Figure 4, Appendix S2). At the beginning of storage, spores of plants #1 and #2 showed germination percentages above 99%. Spores of plant #1 maintained high viability (>80%) for three weeks, then declined sharply and lost all viability after seven weeks of storage, with a deterioration rate of 0.85 probit/week and a longevity (*p*_50_) of 3.78 ± 0.12 weeks (Table 1). Spores of plant #2 retained high viability for only one week, losing viability by week 6, with a faster deterioration rate (0.94 probit/week) and shorter *p*_50_ (2.47 ± 0.15 weeks). Spores of plant #3 had the lowest initial germination (66.0%), losing viability by week 3, with the highest deterioration rate (1.23 probit/week) and shortest *p*_50_ (0.38 ± 0.01 weeks). Spores of plant #4, with an initial germination of 88.5%, showed the lowest deterioration rate (0.66 probit/week) but a *p*_50_ of 2.10 ± 0.11 weeks due to lower initial viability.

**Figure 4:**
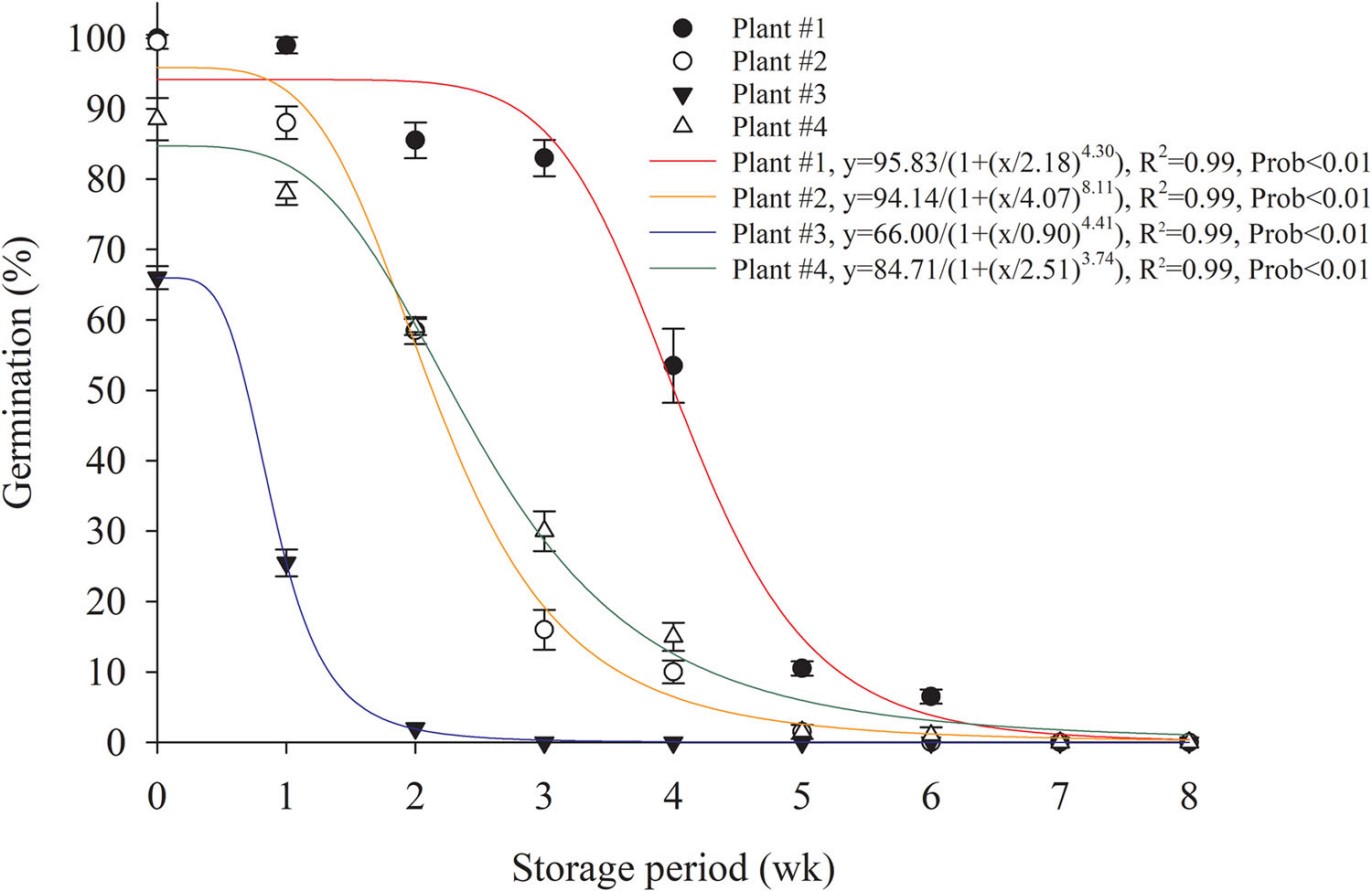
Germination percentages of *Plenasium banksiifolium* spores from four individual plants after different storage periods. Subsamples were stored for 0–8 weeks, and germination was assessed four weeks after sowing each subsample. Error bars represent standard deviation (*n* = 4 spore subsamples per plant). Different letters at week 0 indicate significant differences in initial germination among sources based on one‐way ANOVA with Tukey's HSD test (*p* < 0.05).

**Table 1:** Probit‐transformed initial germination (*K*_i_), deterioration rate (slope), and longevity (*p*_50_) of *Plenasium banksiifolium* spores from four individual plants.

Source	*K*_i_	Slope (1/σ)	*p*_50_ (wk)
Plant #1	3.22	0.85	3.78
Plant #2	2.32	0.94	2.47
Plant #3	0.47	1.23	0.38
Plant #4	1.38	0.66	2.10

### The relative EC ratio (EC_i_/EC_0_) is negatively correlated with germination percentage

A significant negative linear correlation was observed between germination percentage (in probit) and the EC relative ratio (EC_i_/EC_0_) where EC_i_ represents the electrical conductivity measured at time point *i* and EC_0_ represents the initial electrical conductivity (Figure 5). The slopes varied among sources (plant #1 = 2.60, plant #2 = 3.04, plant #3 = 1.99, and plant #4 = 1.55). At 50% germination (0 probit), estimated EC_i_/EC_0_ ratios ranged from 1.38 to 2.11, with a mean of 1.86 ± 0.33 (*n* = 4). At 0% germination (−3.09 probit), EC_i_/EC_0_ ratios ranged from 2.91 to 4.05, with a mean of 3.30 ± 0.53 (*n* = 4) (Table 2).

**Figure 5:**
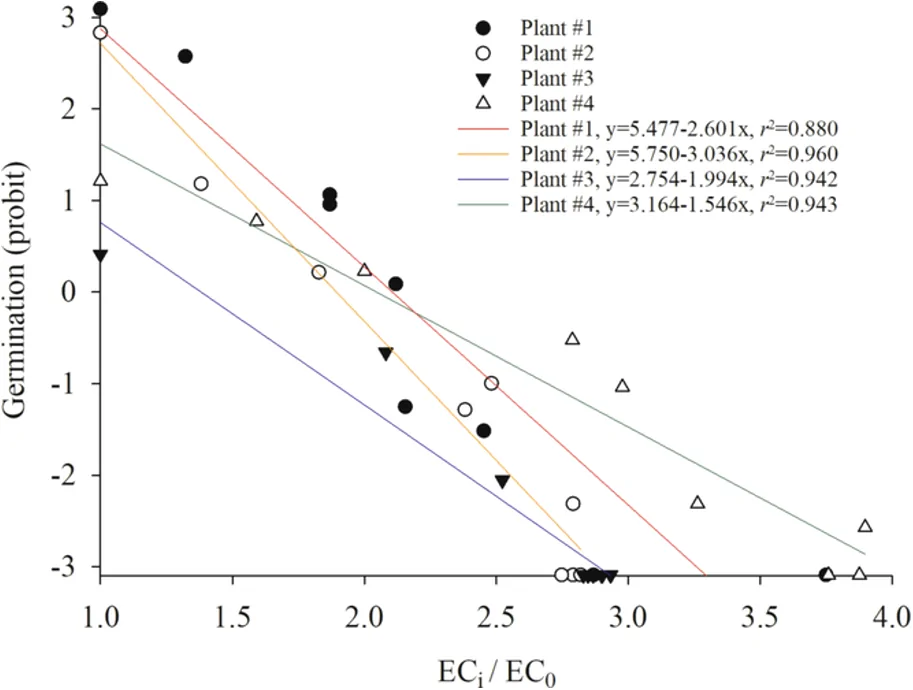
Relationship between germination and relative electrical conductivity (EC_i_/EC_0_) in *Plenasium banksiifolium* spores. Germination percentages, expressed in probit units, were plotted against the EC_i_/EC_0_ ratio for spores from four individual plants. Lines indicate fitted linear regressions for each source.

**Table 2:** Estimated relative electrical conductivity ratios (EC_i_/EC_0_) corresponding to germination thresholds of 50% and 0% in *Plenasium banksiifolium* spores from four individual plants.

Plant source	EC_i_/EC_0_ ratio at 50% germination	EC_i_/EC_0_ ratio at 0% germination
Plant #1	2.11	3.29
Plant #2	1.89	2.91
Plant #3	1.38	2.93
Plant #4	2.05	4.05
Mean ± SD	1.86 ± 0.33	3.30 ± 0.53

## DISCUSSION

### Applicability of EC testing to fern spores

The EC test, which measures electrolyte leakage during immersion, is a well‐established method for assessing seed quality, including viability and vigor (Hibbard and Miller, 1928; Thomas, 1960; ISTA, 2025). In this study, we report the first successful adaptation of the EC test to evaluate the viability and longevity of fern spores, using chlorophyllous spores of *P. banksiifolium* as a model. This adaptation is significant because fern spores represent a different stage in the plant life cycle and are structurally distinct from seeds: they are unicellular, much smaller, and thus require modifications to standard EC protocols.

Unlike standard seed EC protocols, which typically require soaking 50 seeds for 24 h (Matthews and Powell, 2006), our optimized method used only 0.01 g of spores and a 1‐h immersion period. This efficiency may stem from the small size of spores which, compared with seeds, confers a higher surface area/volume ratio and may facilitate rapid ion diffusion and equilibration. This is consistent with Fick's laws of diffusion, wherein the total rate of exchange increases with the surface area available for diffusion (Fick, 1855; Nobel, 2009). This study demonstrates that highly sensitive microbalances and handheld EC meters are practical and reliable measurement tools for such microscale applications. Fern spores are broadly categorized as chlorophyllous or non‐chlorophyllous based on pigment content and wall thickness (Lloyd and Klekowski, 1970). Chlorophyllous spores, such as those of *P. banksiifolium*, have thinner walls, potentially enabling faster electrolyte leakage compared to non‐chlorophyllous spores, which typically possess thicker walls that may slow ion release. We hypothesize that non‐chlorophyllous spores may require longer immersion periods to reach osmotic equilibrium, suggesting that EC testing protocols may require adjustments according to spore type. Further studies involving a broader range of fern taxa are needed to validate this approach and establish standardized EC protocols for different spore types.

### Interpreting EC variation across spore sources

Significant variation in EC_0_ was observed among fresh spores from different *P. banksiifolium* plants, with up to a fivefold difference (Figure 3, plant #3 vs. plant #1). Our data suggest that EC_0_ could serve as a novel proxy for spore quality, with lower values indicating higher longevity (Table 1). This variability likely reflects differences in spore maturity, as observed in seeds where conductivity decreases with advancing ripeness (Cruz‐Tejada et al., 2018). In ferns, spore maturity is a critical factor influencing germination viability and vigor, with immature spores exhibiting lower germination percentages, longer germination times, reduced longevity, and sometimes abnormal gametophyte development compared to mature ones (Mikula et al., 2009; Huang et al., 2024a). The superior performance of mature spores may reflect metabolic reprogramming and reserve accumulation that reduce cytoplasmic electrolyte concentrations, as well as strengthened membranes and walls that reduce passive ion leakage (Kumar and Mishra, 2014; Amir et al., 2018; Sripathy and Groot, 2023).

This study also provides insights into the aging process of fern spores, an area that remains underexplored. Earlier studies have investigated mechanisms of viability loss, with Beri and Bir (1993) demonstrating that aging in *Pteris vittata* L. is linked to the depletion of soluble substances and reduced water uptake. Our research builds on these findings by showing that EC values increase over time across all samples (Figure 3), concomitant with decreased germination percentages, suggesting that electrolyte leakage from the spore protoplasm could be a factor in viability loss during storage. Notably, although absolute EC values varied across sources, the relative EC ratio (EC_i_/EC_0_)—i.e., the EC at time point *i* relative to its initial value—was consistently and significantly negatively correlated with germination percentage (Figure 5). This ratio‐based metric thus normalizes inter‐individual variation and provides a robust indicator for monitoring viability decline in stored spores. Such a relationship could serve as a practical threshold‐based guideline for managing fern spore storage in ex situ conservation efforts.

### Implications for spore bank conservation

Spore banking is an emerging conservation strategy that offers low‐cost, high‐density storage for fern germplasm (Dyer, 1994; Pence, 2004; Ballesteros, 2008). However, traditional germination tests for assessing spore viability are time consuming, often requiring weeks to months, and are impractical for large‐scale screening (Lloyd and Klekowski, 1970; Ballesteros and Walters, 2011).

Our study establishes the EC test as a rapid, reproducible alternative for assessing spore quality. The method requires only a 1‐h assay and a small amount of material (0.01 g), making it suitable for large‐scale screening (Figure 6A). We further show that fresh spores with lower EC_0_ values exhibit higher initial viability and greater longevity, confirming the predictive power of the test (Table 1, Figures 3 and 4, conceptualized in Figure 6B). Our findings also suggest practical thresholds for spore bank monitoring: an EC_i_/EC_0_ ratio of ~2 corresponds to a 50% decline in germination, while a ratio of ~3 indicates complete loss of viability (Table 2, Figure 5, conceptualized in Figure 6C). These thresholds offer actionable criteria for identifying aging spore lots that may require regeneration or replacement.

**Figure 6:**
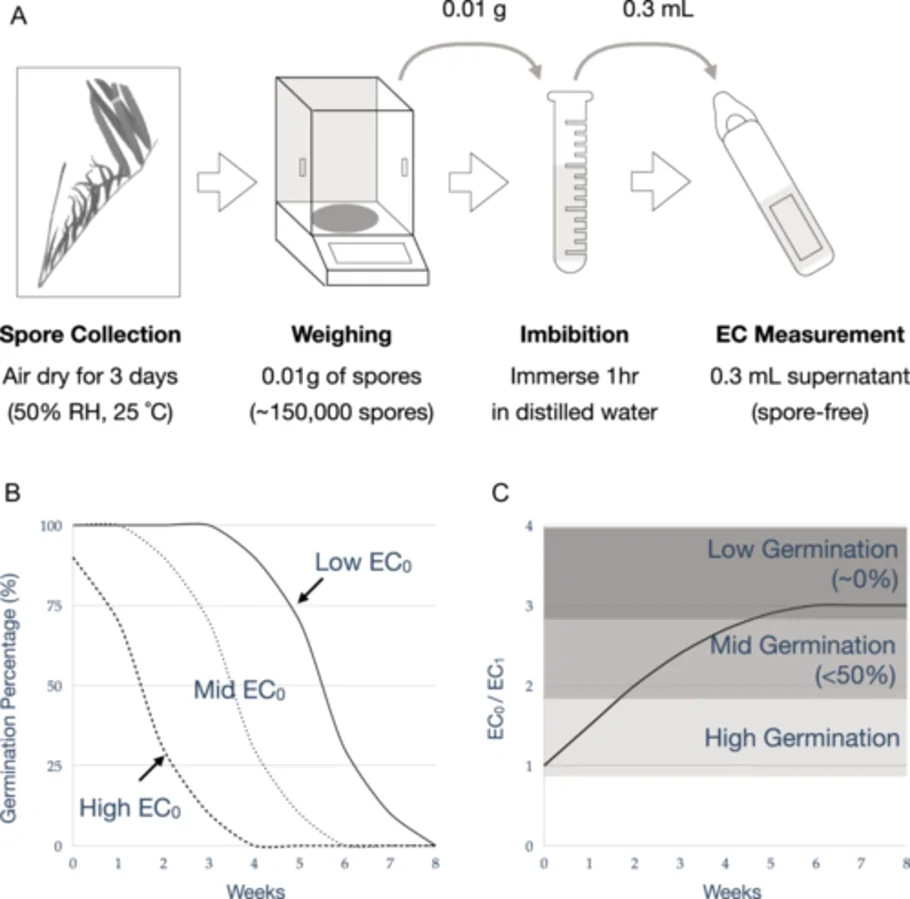
Summary of EC test application for assessing chlorophyllous fern spore vigor. (A) Schematic workflow of the optimized EC test protocol for *Plenasium banksiifolium* spores. (B) Conceptual model illustrating the relationship between initial EC value (EC_0_) and spore quality: spores with lower EC_0_ exhibit higher initial viability and longer longevity. (C) Viability thresholds inferred from relative EC ratio (EC_i_/EC_0_): spores approach 50% germination loss when EC_i_/EC_0_ ≈ 2.0 and complete loss of viability when EC_i_/EC_0_ ≈ 3.0. RH, relative humidity.

Based on our initial validation of the EC test using chlorophyllous spores of *P. banksiifolium*, we propose the following guidelines for spore bank management: (1) measure EC_0_ when a new batch of fresh spores is collected and consider discarding batches that exhibit higher‐than‐average EC_0_ values, as such spores are likely to have reduced storage longevity (Figure 6B); and (2) remeasure EC when viability loss is suspected, because EC_i_/EC_0_ shows a negative correlation with germination percentage (Figure 5). When the EC_i_/EC_0_ ratio exceeds approximately 2, regeneration through sowing should be considered before complete loss of viability occurs (Table 2).

However, this proof‐of‐concept study has limitations. A primary constraint concerns the extension of this method to non‐chlorophyllous spores, which lack chlorophyll in mature spores and typically possess thicker perispores, higher lipid contents, and greater antioxidant activity (Lloyd and Klekowski, 1970; Ballesteros et al., 2018, 2019). These traits enable them to remain viable for years or even decades, even under herbarium storage at room temperature (Johnson, 1985; Windham et al., 1986), making them well suited for spore banking. Their longevity, however, complicates the establishment of EC response curves, as this would require measuring EC_0_ in freshly collected material and conducting long‐term storage experiments until viability is largely lost. One possible strategy would be to infer the relationships among EC, germination percentage, and age using archived spore lots collected across different years. However, such an approach assumes comparable initial vigor among batches and a simple relationship between germination percentage decline and EC values. Our results indicate that these assumptions are unlikely to hold. We observed substantial variation in longevity among morphologically indistinguishable batches (Figure 4), and germination percentage showed a stronger relationship with the EC_i_/EC_0_ ratio rather than with EC_0_ alone (Figure 5). These findings are consistent with our previous observations of cryptic variation in spore maturity (Huang et al., 2024a) and with the non‐linear relationship between germination and longevity observed in this study (Figure 4). Thus, while applying EC testing to non‐chlorophyllous spores remains an important and promising direction, meaningful validation would require substantial spore material, carefully controlled storage experiments, and repeated EC measurements spanning the full decline curve from fresh spores to near‐total loss of viability. Such long‐term studies extend beyond the scope of the present work but will be essential for further assessing the generality of this protocol. Intermediate spore types may provide a practical starting point. For example, *Matteuccia struthiopteris* (L.) Tod. produces chlorophyllous spores with greater desiccation tolerance, increased hydrophobicity, and longer longevity compared with other chlorophyllous spores (López‐Pozo et al., 2019). Likewise, “cryptochlorophyllous” spores found in *Elaphoglossum* Schott ex J.Sm. (Dryopteridaceae), *Lomariopsis* Fée (Lomariopsidaceae), and *Pleopeltis* Humb. & Bonpl. ex Willd. (Polypodiaceae) contain chlorophyll but possess thicker perispores and higher concentrations of pigments or storage compounds, giving them a non‐green appearance (Sundue et al., 2011). These taxa may therefore serve as useful model systems for extending EC testing across a broader range of spore physiological types.

### Broader ecological and conservation applications

The EC test shows strong promise as a sensitive indicator of spore quality, particularly in detecting subtle differences in spore maturity that are not readily distinguishable morphologically but strongly influence germination rate and storage longevity. The ability of the EC test to detect these hidden differences highlights its practical value for germplasm conservation.

In addition, given its rapid detection capability, the spore EC test may serve as an additional ecotoxicity indicator based on electrolyte leakage, complementing mitochondrial activity–based TTC assays (Catala et al., 2009; Gabriel y Galán and Prada, 2010).

The EC test may also be applicable to spores that are dormant (e.g., *Isoetes* L.; Huang et al., 2024b) or have complex germination requirements, such as those involving mycorrhizal associations, in which conventional germinating percentages are not indicative of the intrinsic viability of spores (Lehnert and Kessler, 2016; Pedrero‐López et al., 2021). In such cases, EC testing can help screen for viable spores before investing in germination trials and culture optimization.

### Conclusions

This study demonstrates the feasibility and utility of the EC test for assessing the quality of chlorophyllous fern spores. Its adaptation for microscale samples and short soaking periods makes it particularly suitable for the unique characteristics of fern spores. Future research should focus on validating this method across a broader range of fern species, including both chlorophyllous and non‐chlorophyllous types, and on elucidating the mechanisms behind the observed correlations between EC, germination, and longevity. The integration of EC testing into standard protocols for spore banks could significantly enhance the efficiency and effectiveness of fern conservation efforts, paving the way for more robust and sustainable spore banking practices.

## AUTHOR CONTRIBUTIONS

Y.‐M.H. and W.‐L.C. conceived and designed the research. C.‐W.K. collected the data. Y.‐M.H., C.‐T.D., C.‐M.H., W.‐L.C., and T.‐T.K. analyzed and interpreted the data. Y.‐M.H. and T.‐T.K. drafted the manuscript. All authors reviewed and approved the final version of the manuscript.

## Supporting information


**Appendix S1:** Pretest for spore electrical conductivity (EC).

**Appendix S2:** Serial electrical conductivity measurements and germination testing of *Plenasium banksiifolium* spores.

## Data Availability

The raw data for this study (electrical conductivity and germination test results) are provided in the Supporting Information.
